# Modeling the Hydrogen Redistribution at the Grain Boundary of Misoriented Bicrystals in Austenite Stainless Steel

**DOI:** 10.3390/ma15020479

**Published:** 2022-01-09

**Authors:** Fuqiang Yang, Tao Yan, Wenjuan Zhang, Haibing Zhang, Lingyan Zhao

**Affiliations:** 1 School of Science, Xi’an University of Science & Technology, Xi’an 710054, China; zhaolingyan@xust.edu.cn; 2 School of Mechanical Engineering, Xi’an University of Science & Technology, Xi’an 710054, China; 19205201065@stu.xust.edu.cn (T.Y.); 19205201077@stu.xust.edu.cn (W.Z.); 3 State Key Laboratory for Marine Corrosion and Protection, Luoyang Ship Material Research Institute (LSMRI), Qingdao 266237, China; zhanghb@sunrui.net

**Keywords:** crystal orientation, bicrystal model, hydrogen diffusion, diffusion model, finite element method

## Abstract

Hydrogen embrittlement, as one of the major concerns for austenitic stainless steel, is closely linked to the diffusion of hydrogen through the grain boundary of materials. The phenomenon is still not well understood yet, especially the full interaction between hydrogen diffusion and the misorientation of the grains. This work aimed at the development of a robust numerical strategy to model the full coupling of the hydrogen diffusion and the anisotropic behavior of crystals in 316 stainless steel. A constitutive model, which allows easy incorporation of crystal orientation, various loading conditions, and arbitrary model geometries, was established by using the finite element package ABAQUS. The study focuses on three different bicrystal models composed of misoriented crystals, and the results indicate that the redistribution of hydrogen is significant closely to the grain boundary, and the redistribution is driven by the hydrostatic pressure caused by the misorientation of two neighboring grains. A higher elastic modulus ratio along the tensile direction will lead to a higher hydrogen concentration difference in the two grains equidistant from the grain boundary. The hydrogen concentration shows a high value in the crystal along the direction with stiff elastic modulus. Moreover, there exists a large hydrogen concentration gradient in a narrow region very close to the grain boundary to balance the concentration difference of the neighboring grains.

## 1. Introduction

The hydrogen embrittlement (HE), which is associated with the trapping and diffusion of aggressive hydrogen in metals under stress, could drastically reduce the expected ductility and toughness of steels and result in catastrophic failures [1,2]. According to the macroscopic property of HE, three main mechanisms have been proposed: (1) the hydrogen enhanced decohesion (HEDE) [3,4], (2) the hydrogen-enhanced localized plasticity (HELP) [5,6], and (3) the adsorption-induced dislocation emission (AIDE) [7,8]. Despite much research into the issue of HE [2], the topic is still widely debated. The contribution of the grain boundaries (GBs) to the diffusion and trapping of hydrogen remains a controversial point in the understanding of hydrogen embrittlement phenomena [9].

As a discontinuous region, the grain boundaries often act as barriers to plastic flow or the sources of slip and dislocation, these physical structures could affect the hydrogen diffusion. Several studies [10,11,12,13] have confirmed that hydrogen diffusion is accelerated along the GBs by a mechanism of short-circuit diffusion. Even though Louthan et al. [14] reported that the acceleration of hydrogen diffusion along GBs is caused by the geometrically necessary dislocations (GNDs) stored in these interfaces. Ladna and Birnbaum [15,16] associate the boundary energy with the diffusion, and confirmed that hydrogen diffusion is accelerated along tilt high-energy boundaries and that in low-energy boundaries the hydrogen diffusion stays the same as in the lattice.

On the contrary, suppressed diffusion of hydrogen at grain boundaries was found. Yao and Canoon [17] argued that there are more dislocations and vacancies stored in the grain boundaries as trapping sites which impedes the diffusion, and competition between the short-circuit diffusion and hydrogen trapping occurs at the grain boundary. Ichimura [18] also supported this view and pointed out that the suppressed diffusion might be remarkable for the sample with small grain size

While Mütschele and Kirchheim [19] concluded that the diffusion coefficient depends on the hydrogen concentration, the GBs impede diffusion at low concentrations, and provide a fast pathway for diffusion at high concentrations. As to the twist boundary, Szpunar et al. [20] simulated the classical dynamics of a hydrogen atom in the vicinity of twin–twist GBs, and concluded that the diffusivity is enhanced at these GBs. However, Pedersen et al. [21] argued that the twist boundary turns out to block diffusion across the boundary, only the diffusion parallel to the boundary is slightly enhanced because of the reduced configuration space.

Nagao et al. [22] have confirmed that the hydrogen diffusion and accumulation is significantly promoted by an applied stress field even in the steel with traps for hydrogen. By assuming the local equilibrium of hydrogen in traps and normal interstitial lattice sites [23], the effects of the hydrostatic stress and trapping on the hydrogen distribution in plastically deforming steel were studied [24,25,26], and the results indicate that the total hydrogen concentration and plastic strain decreases with distance from the crack tip whereas the hydrostatic stress rises.

Due to elastic anisotropy, the anisotropic elastic behavior of microstructure at grain boundaries can produce local stress concentrations [27] and strong hydrostatic stress gradients under mechanical loading even without notch or crack [28,29]. In the present work, the hydrogen diffusion at the grain boundary was investigated by a proposed hydrogen diffusion model, which is based on the elastic response of the anisotropic behavior of crystals. The effects of crystal orientation induced stress–strain heterogeneity on the hydrogen redistribution in stainless steel 316 L polycrystals was estimated

## 2. Governing Equations of Hydrogen Diffusion

The diffusion phase should satisfy the law of conservation of mass during diffusion, namely

(1) $$\int_{V}\frac{\partial c}{\partial t}dV+\int_{S}\vec{n}\cdot \vec{J}dS=0$$

where *c* is the mass concentration of hydrogen in steel; *V* is any volume whose surface is *S*; $\vec{n}$ is the outward normal to *S*; $\vec{J}$ is the flux of concentration of the diffusing phase; $\vec{n}\cdot \vec{J}$ is the concentration flux leaving *S* surface.

The diffusion of hydrogen in heterogeneous steel can be given by the extended Fick’s law, which considers that the flux is proportional to the gradient of chemical potential [30,31],

(2) $$J=-\frac{Dc}{RT}\nabla u$$

where *D* is the diffusivity; *R* is the universal gas constant, 8.3144 J·mol^−1^·K^−1^; *T* is the absolute temperature, K; *u* is the chemical potential. For a system under constant pressure and temperature, the chemical potential is given as [24],

(3) $$u=u_{\sigma }+RT\ln\phi +\sigma_{h}V_{H}$$

where $\mu_{\sigma }$ is the stress dependent part of the chemical potential; *σ*_h_ is the hydrostatic pressure, which is calculated by the diagonal terms of the stress tensor, *σ*_h_ = −∑σ*_ii_*/3; *V*_H_ represents the partial molar volume of hydrogen; *ϕ* is the hydrogen concentration in steel normalized by its solubility *s* with ϕ=c/s.

By substituting Equation (3) into (2) and considering the normalized concentration, the hydrogen flux is derived as

(4) $$J=-sD\cdot \nabla \phi -sDk_{\sigma }\cdot \nabla \sigma_{h}$$

where *k_σ_* represents the pressure stress factor, which governs the mass diffusion driven by the gradient of the equivalent pressure stress. It is defined as a function of concentration and temperature,

(5) $$k_{\sigma }=\frac{V_{H}\phi }{RT}$$

According to Equation (4), there are two driving forces of hydrogen transport. The hydrogen concentration drives the hydrogen diffuse from the high concentration region to the low, and the hydrostatic pressure will drive the hydrogen diffuse from the low pressure region to high.

## 3. Crystallographic Constitutive Model

The relationship between stress and strain of a polycrystalline material, in which the elastic properties of a material depend on its orientation, could be described by the generalized Hooke’s law [32],

(6) $$\sigma_{i}=C_{ij}\varepsilon_{j}$$

(7) $$\varepsilon_{i}=S_{ij}\sigma_{j}$$

where *σ_i_* and *ε_j_* represent the stress components and strain components, respectively; *C_ij_* and *S_ij_* are the stiffness and compliance matrices, with $S_{ij}=C_{ij}^{-1}$ (*i*, *j* = 1, 2, …, 6).

As the different crystal systems can be characterized exclusively by their symmetries, the elastic constants could be reduced. In a cubic system, there are three mutually perpendicular axes of symmetry, and the elastic constants could be reduced to three along different axes, and the stiffness matrices is,

(8) $$C=\left[\begin{array}{cccccc} c_{11} & c_{12} & c_{13} & & & \\ c_{21} & c_{22} & c_{23} & & & \\ c_{31} & c_{32} & c_{33} & & & \\ & & & c_{44} & & \\ & & & & c_{55} & \\ & & & & & c_{66} \end{array}\right]$$

in which the elastic constants are *C*_11_ = *C*_22_ = *C*_33_, *C*_44_ = *C*_55_ = *C*_66_, and *C*_12_ = *C*_13_ = *C*_23_ = *C*_21_ = *C*_31_ = *C*_32_.

The elastic modulus *E*, Poisson’s ratio *v*, and shear modulus *G* in a cubic crystal could be achieved as [32],

(9) $$E=\frac{1}{S_{11}}$$

(10) $$\nu =-\frac{S_{12}}{S_{11}}$$

(11) $$G=\frac{1}{S_{44}}$$

where,

(12) $$S_{11}=\frac{C_{11}+C_{12}}{\left(C_{11}-C_{12}\right)\cdot \left(C_{11}+2C_{12}\right)}$$

(13) $$S_{12}=\frac{-C_{12}}{\left(C_{11}-C_{12}\right)\cdot \left(C_{11}+2C_{12}\right)}$$

(14) $$S_{44}=\frac{1}{C_{44}}$$

In a cubic material, the elastic moduli can be determined along any orientation, from the elastic constants, by application of the following equation,

(15) $$\frac{1}{E_{ijk}}=S_{11}-2k\left(S_{11}-S_{12}-\frac{1}{2}S_{44}\right)$$

(16) $$k=l_{i1}^{2}l_{j2}^{2}+l_{j2}^{2}l_{k3}^{2}+l_{k3}^{2}l_{i1}^{2}$$

where *E_ijk_* is the Young’s modulus, respectively, in the [*ijk*] direction; *k* is the orientation coefficient; *l_i_*_1_, *l_j_*_2_, and *l_k_*_3_ are the direction cosines of the direction [*ijk*].

## 4. Finite Element Model

### 4.1. Geometry Model

**Figure 1:**
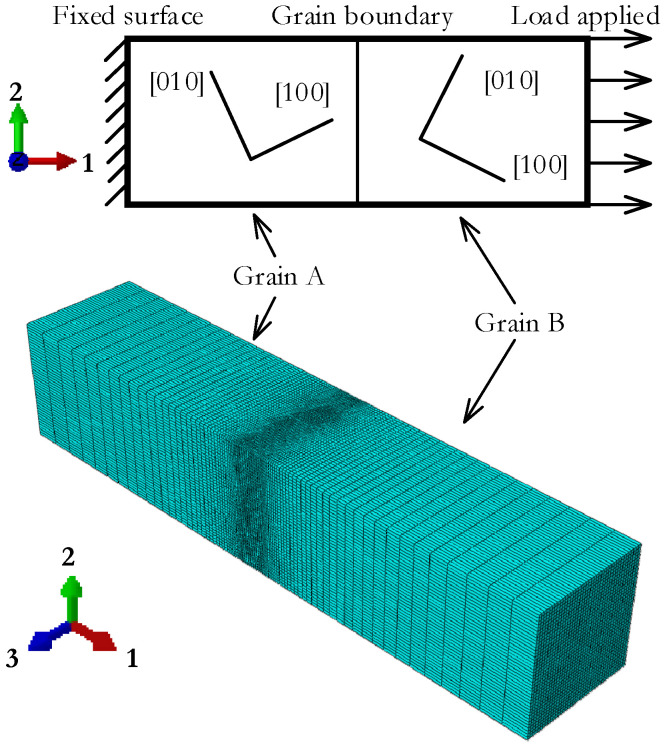
Geometry model of the bicrystal.

A bicrystal model was constructed with different grain orientations, as shown in Figure 1. The average grain size of 316 L stainless steel varies from 17 μm to 200 μm with different aging time, temperatures, and other factors in the literature [33,34,35]; the dimension of each component grain in the model is assumed to be 20 μm × 20 μm × 50 μm.

### 4.2. Material Model

**Figure 2:**
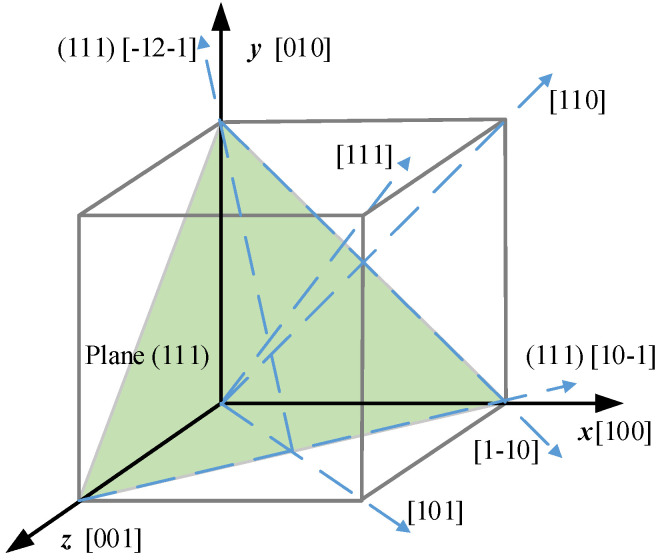
The selected crystallographic direction.

316 stainless steel is a polycrystalline aggregate and is randomly oriented, the material is macroscopically isotropic. However, the individual grain has a face-centered cubic (FCC) structure and exhibits crystalline anisotropy and symmetry. The elastic constants of a 316 stainless steel crystal are *C*_11_ = 204.6 GPa, *C*_12_  = 137.7 GPa, and *C*_44_ = 126.2 GPa [36]. Substituting the elastic constants into Equations (9)~(14), the elastic modulus *E*, shear modulus *G*, and Poisson’s ratio *ν* are 93.8 GPa, 126.2 GPa, and 0.40 respectively. According to Equations (16) and (17), the elastic modulus along the <110> and <111> directions are 193.6 GPa and 299.8 GPa, respectively. The <100> direction is softer whereas the <111> direction is stiffer, and *E*_111_ > *E*_110_ > *E*_100_ = *E*, as shown in Figure 2.

**Figure 3:**
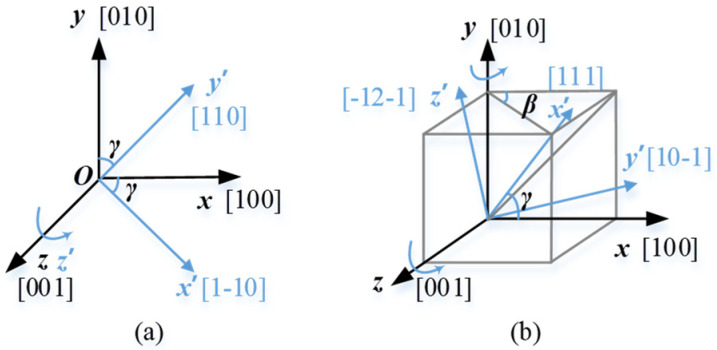
Grain formation process: (**a**) MID grain, and (**b**) ST grain.

Considering the relationship between the [100], [110], and [111] orientations and the stretching axis, three types of crystals with different orientations were modeled [28], as shown in Table 1. The most compliant grain (MC) has the coordinate axes *x′*, *y′*, and *z′* of the local coordinates, which represent the crystal orientations [100], [010], and [001] respectively, and it is consistent with the global coordinate *o-xyz.* As shown in Figure 3a, the local coordinate of the intermediate stiffness grain (MID) has the (001)-[1-10] direction as the axis *x′* and the (001)-[110] direction as *y′*, by rotating the grain about the [001] direction with angle *γ*, the MID grain has its local coordinate consistent with the global coordinate. By defining the (111)-[10-1] and (111)-[-12-1] directions as the crystal axes *y′* and *z′*, the stiffness grain (ST) has the [111] direction as the axes *x′*, and the local coordinate of the ST grain will coincide with the global coordinate by rotating the local coordinate about the axis *y* and *z* with angle *β* and *γ* in sequence. Among the three different grain types, the planes perpendicular to the global coordinate axis *x* are the (100), (110), and (111) planes in the MC, MID, and ST grains, respectively.

As shown in Figure 3, to ensure the material coordinate *o-x′y′z′* coincide with the global coordinate *o-xyz*, the material coordinate *o-x′y′z′* should be rotated. The rotation matrices of material coordinate *o-x′y′z′* with respect to the global coordinate *o-xyz* are [37],

(17) $$R_{x}\left(\alpha \right)=\left[\begin{array}{ccc} 1 & 0 & 0\\ 0 & \cos\alpha & -\sin\alpha \\ 0 & \sin\alpha & \cos\alpha \end{array}\right]$$

(18) $$R_{y}\left(\beta \right)=\left[\begin{array}{ccc} \cos\beta & 0 & \sin\beta \\ 0 & 1 & 0\\ -\sin\beta & 0 & \cos\beta \end{array}\right]$$

(19) $$R_{z}\left(\gamma \right)=\left[\begin{array}{ccc} \cos\gamma & -\sin\gamma & 0\\ \sin\gamma & \cos\gamma & 0\\ 0 & 0 & 1 \end{array}\right]$$

where *α*, *β*, and *γ* are the rotation angles about the axis *x*, *y*, and *z*, respectively, with counter-clockwise positive.

Thus, a vector having a composition rotation about the axes *x*, *y*, and *z* in sequence could be expressed as,

(20) $${\vec{p}}_{\mathrm{new}}=R\cdot {\vec{p}}_{\mathrm{old}}$$

where ${\vec{p}}_{\mathrm{new}}$ is the new vector of ${\vec{p}}_{\mathrm{old}}$ after the composition rotation, both of them are in the same coordination frame of *o*-*xyz*; *R* is the composition rotation matrix as,

(21) $$R=R_{z}\left(\gamma \right)R_{y}\left(\beta \right)R_{x}\left(\alpha \right)$$

With the three different types of single crystal, three incompatible bicrystal models were constructed, as shown in Table 2.

Besides the mechanical properties, the diffusivity and solubility of hydrogen in steel are given by Fujii et al. as [38],

(22) $$s=4300e^{-3261/T}$$

(23) $$D=\frac{7611\times 10^{-5}e^{-1157/T}}{1+\left(1.05\times 10^{-3}e^{-3573/T}\right)}$$

### 4.3. Boundary Conditions, Loading Process, and Meshing

In the finite element model, the surface perpendicular to direction 1 of grain A is fixed initially, and then a constant displacement U_1_ equal to 0.5 is applied to the right surface of grain B. The simulation temperature is assumed to be 325 K, and the diffusivity and solubility of hydrogen in steel are calculated as 3.4096 × 10^−5^ mm^2^·s^−1^ and 0.1887 ppm mm·N^−1/2^. With a partial molar volume of hydrogen *V*_H_ in the iron-based metal equals 2.0 × 10^3^ mm^3^·mol^−1^, the pressure stress factor *k_σ_* is 3.92189. It is assumed that the initial hydrogen concentration in the whole model is 50 ppm, which corresponds to a normalized concentration of 265 N^1/2^·mm^−1^.

When establishing a finite element model that can be directly used in simulation calculations, the quality of the finite element mesh is the main consideration in determining the calculation scale and calculation accuracy. The focus of this paper is the distribution of hydrogen concentration near the grain boundary. To balance the calculation scale and calculation accuracy during grid division, a denser grid is divided near the grain boundary and a sparse grid is divided in other places to ensure a short calculation time and good calculating accuracy. The element type used is DC3D8R.

## 5. Results and Discussion

### 5.1. The Hydrogen Concentration Distribution in the Model I Crystal

**Figure 4:**
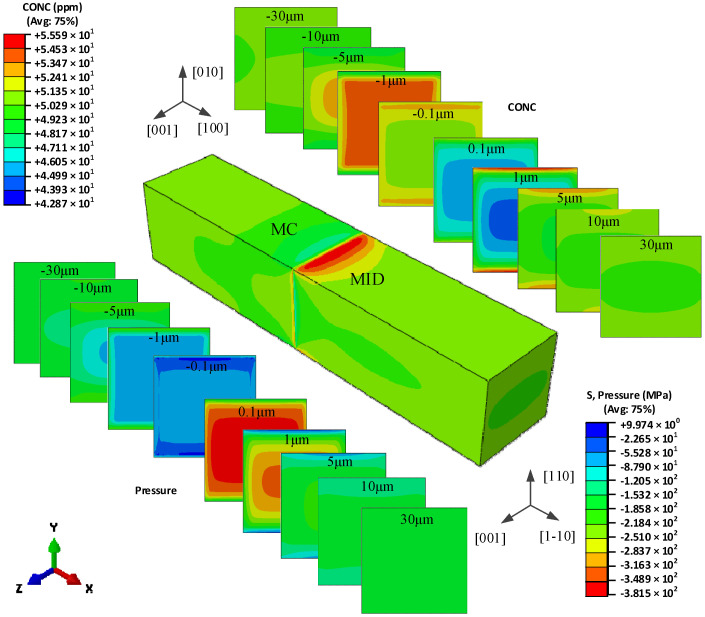
The hydrogen concentration distribution in the model I bicrystal.

Figure 4 shows the redistribution of hydrogen in the global model of type I bicrystal, and the hydrogen concentration distribution in different cross-sections of the two different grains. The orientation mismatch of the two grains causes a significant hydrogen difference near the grain boundary. The cross-sections of the two grains, which respect the (100) plane and (1-10) plane in MC grain and MID grain, respectively, have different hydrogen concentration distributions.

On the cross-section 0.1 μm apart from the GB in the MID grain, the hydrogen shows a plateau in the middle of the cross-section with low concentration, and increases towards the surface. When the distance increases to 1 μm, the hydrogen concentration decreases a little in the middle and increases at the surface, especially in the *y*-direction, which represents the [110] orientation. With the cross-section having an increased distance from the grain boundary, the hydrogen concentration decreases at the surface and increases in the middle, and eventually has little difference.

In MC grains, the hydrogen concentration is also lower in the middle, larger and increasing toward the surface, however, the maximum concentration seems to be located near the surface in the y direction, representing the [010] orientation. When the distance is increased to −1 μm, the concentration has a larger value, the middle platform is larger, and the surface value is smaller. The hydrogen has an opposite distribution on the cross-section of −0.1 μm and −1 μm away from the grain boundary. With an increased distance from the grain boundary, the hydrogen concentration increases on the surface and decreases in the middle, and eventually has little difference. Whether the distribution of hydrogen in the MC grain or the MID grain, it is symmetrical about the X-Y plane and X-Z plane, and it is similar to the hydrostatic pressure distribution, which proves that the redistribution of hydrogen is driven by the unequal hydrostatic pressure induced by the misorientation of the two grains.

**Figure 5:**
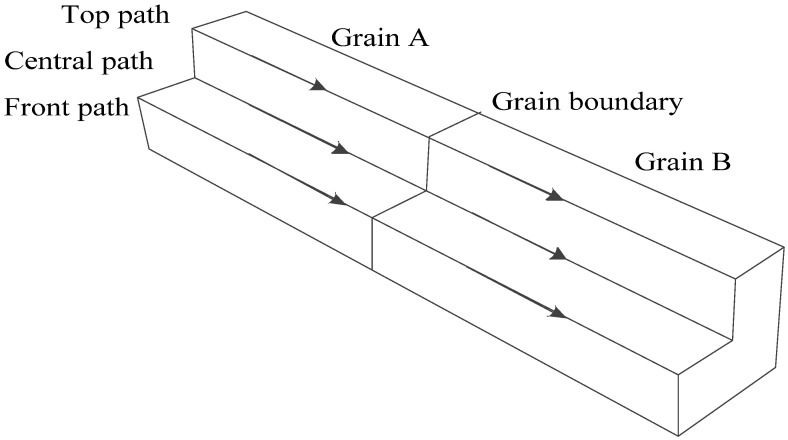
Observation paths.

**Figure 6:**
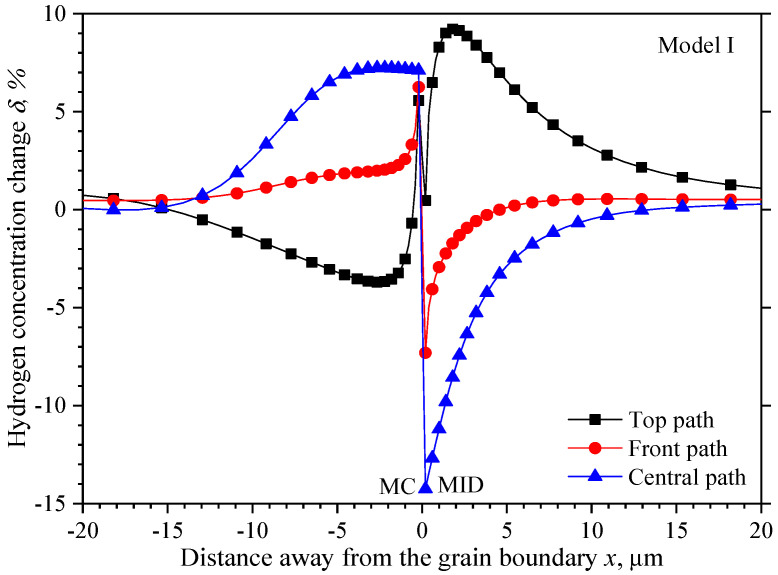
Hydrogen concentration change along observation paths in model I.

A more intuitive hydrogen concentration change *δ* relative to the initial concentration caused by the unequal hydrostatic pressure is observed along the three paths shown in Figure 5, and the results are shown in Figure 6. The research has shown that the stress distribution near grain boundaries depends strongly on the crystal orientation, and the stress is discontinuous in the two misoriented grains on the grain boundary [23]. However, the step hydrogen concentration differences will not happen on the grain boundary due to the identical hydrogen solubility in the two misoriented grains. When the distance is very close to the grain boundary, about ±0.2 μm, the hydrogen concentration differences are apparent in the two grains equidistant from the grain boundary, and a larger gradient exists in the narrow region to ensure the same concentration on the boundary.

Beyond the narrow region, the lowest hydrogen concentration in the MID grain is 0.5%, −7.3%, and −14.3% on the top, front, and central path, respectively. The hydrogen concentration along the front path and central path increases continuously to the initial value when the distance away from the grain boundary, while the hydrogen concentration along the top path increases to the highest value of 9.2% at 2 μm apart from the grain boundary, and then decreases gradually to the initial value. The vertical distance between each two curves presents the hydrogen concentration difference, so the hydrogen away has the highest concentration on the top path and the lowest concentration on the central path. The hydrogen concentration has a larger gradient from the center towards the top than the front. In contrast to the hydrogen concentration in the MID grain, the highest value will be first achieved as 5.6%, 6.3%, and 7.1% on the top, middle, and front path respectively beyond the narrow region. With the distance away from the grain boundary, the hydrogen concentration decreases gradually along the middle path; a short concentration platform with small fluctuations extends to about −4 μm exists on the front path, and then decreases gradually close to the initial value. While the hydrogen concentration will decrease to the lowest value of −3.9% at 2.7 μm, and then increases gradually close to the initial value.

**Figure 7:**
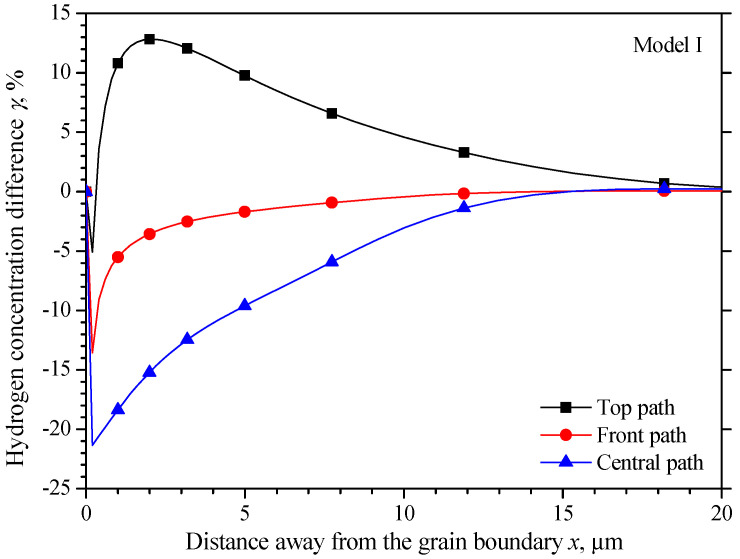
Hydrogen concentration difference equidistant from the grain boundary of model I.

Figure 7 compares the hydrogen concentration difference *γ* in the two different grains equidistant from the grain boundary,

(24) $$\gamma =\delta_{R}-\delta_{L}$$

where *δ_R_* and *δ_L_* respect the hydrogen concentration change in the right and left grains equidistant from the grain boundary, respectively. The maximum hydrogen concentration difference on the middle and front path is −13.6% and −21.4% at 0.2 μm equidistant from the grain boundary, and it is 12.8% on the top path at 2 μm equidistant from the grain boundary. The hydrogen concentration difference *γ* decreases from the highest value to about 0 when far from the grain boundary.

### 5.2. The Hydrogen Concentration Distribution in Model II Crystal

**Figure 8:**
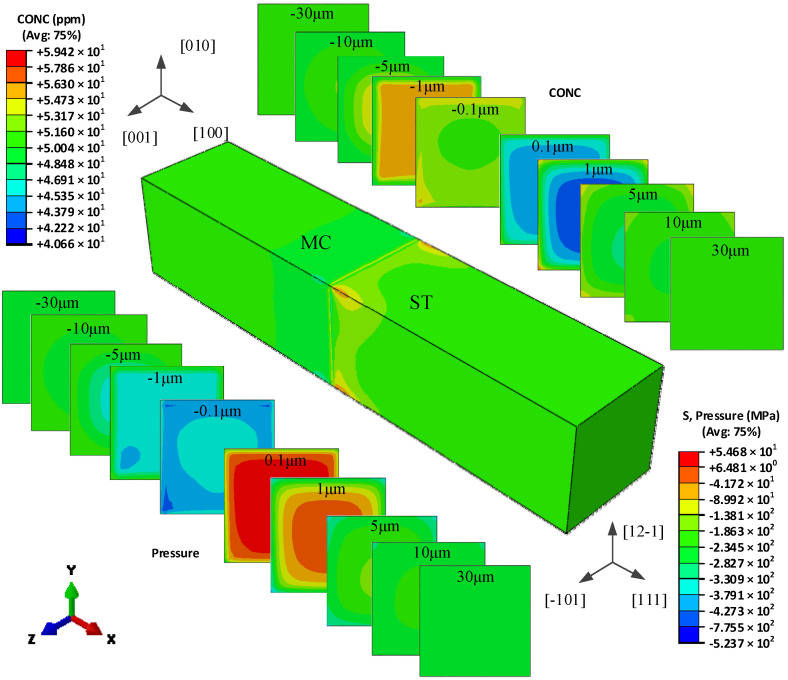
The hydrogen concentration distribution in model II bicrystal.

Figure 8 shows the redistribution of hydrogen in the global model of type II t bicrystal, and the hydrogen concentration distribution in different cross-sections. The highest value seems to locate on the four edges parallel to the *x*-axis in the ST grain. The cross-sections represent the (100) planes in the MC grain, and (-12-1) planes in the ST grain. On the cross-section of both grains, the hydrogen is almost symmetrical about the X-Y plane and X-Z plane, and has the same gradient from the center towards the surface along the *y*-axis and *z*-axis. The obvious distribution could be found on the cross-section within ±5 μm away from the grain boundary, and the differences disappear far from the grain boundary.

**Figure 9:**
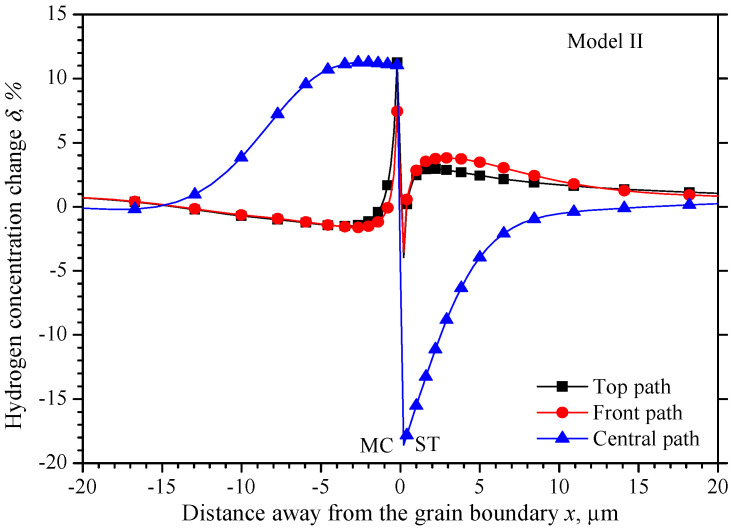
Hydrogen concentration change along observation paths in model II.

Figure 9 shows a more intuitive hydrogen concentration change *δ* along the three observing paths shown in Figure 5. There also exists a narrow region about ±0.2 μm from the grain boundary, in which the hydrogen concentration has a larger gradient from the MC grain to the ST grain. Beyond the narrow region, the lowest hydrogen concentration in the ST grain is −3.9%, −3.5%, and −5.1% on the top, front and central paths, respectively. The hydrogen concentrations on the top path and the front path almost coincide with each other, and they increase to the highest concentration close to 4.5% at 3 μm away from the grain boundary, and then decrease close to the initial value. While the hydrogen increases gradually close to the initial value. The hydrogen concentration distribution on the three paths in the MC grain is similar to that in the ST grain with an opposite trend. The hydrogen concentration on the top path and the front path coincide with each other, but they decrease to the lowest value of −1.5% at 3 μm away from the grain boundary from 2.5%, and then increase close to the initial concentration gradually. On the central path, the hydrogen concentration fluctuates around 11% until −4 μm away from the grain boundary and then decreases close to the initial concentration.

**Figure 10:**
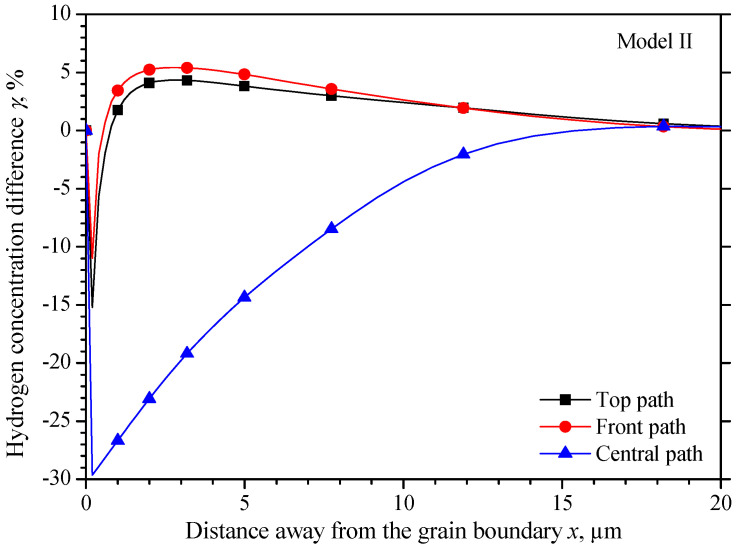
Hydrogen concentration difference equidistant from the grain boundary of model II.

As shown in Figure 10, the maximum hydrogen concentration difference *γ* on the three paths all locate 0.2 μm equidistant from the grain boundary, the values are −15.2%, −10.98%, and −29.6% on the top, front, and central path, respectively.

### 5.3. The Hydrogen Concentration Distribution in Model III Crystal

**Figure 11:**
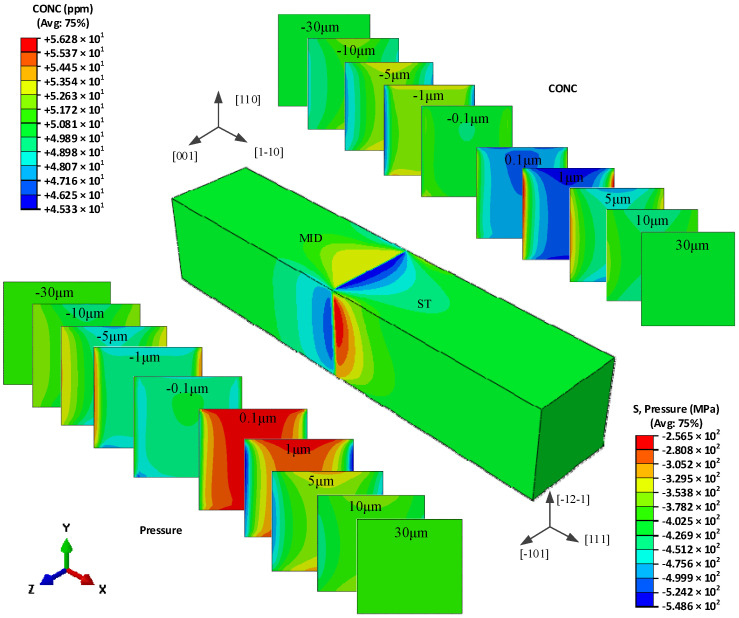
The hydrogen concentration distribution in model III bicrystal.

Figure 11 shows the redistribution of hydrogen in the global model of type III bicrystal, and the hydrogen concentration distribution in different cross-sections. The cross-sections represent the (1-10) planes in the MID grain and (-12-1) planes in the ST grain. On the cross-section of both grains, the hydrogen is almost symmetrical about the X-Y plane and X-Z plane. The obvious distribution could be found on the cross-section within ±5 μm away from the grain boundary, and the differences disappear far from the grain boundary.

**Figure 12:**
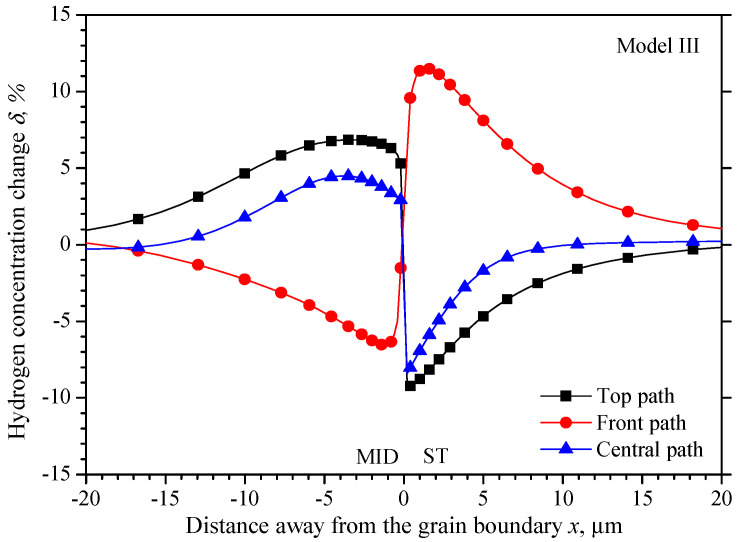
Hydrogen concentration change along observation paths in model III.

Figure 12 shows the hydrogen concentration change *δ* along the three observing paths shown in Figure 5. A larger hydrogen concentration gradient locates within the ±0.2 μm over the grain boundary to ensure the two grains have consistent hydrogen concentration. In the ST grain, the hydrogen concentration on the top path and the central path increases gradually from −9.0% and −8.4% at 0.2 μm to the initial value with the increasing distance. While the hydrogen concentration on the front path will increase from 6.0% at 0.2 μm to the highest value of 11.5% at 1.4 μm, and then decrease gradually to the initial value. In the MID grain −0.2 μm beyond the grain boundary, the hydrogen concentration will increase firstly from 5.3% and 2.9% to 6.8% and 4.5% at −4 μm away from the grain boundary on the top and central path, then decreases gradually. While the hydrogen concentration on the front path decreases from −1.5% to the lowest value of −6.5% at −1.4 μm away from the grain boundary and then increases gradually to the initial value.

**Figure 13:**
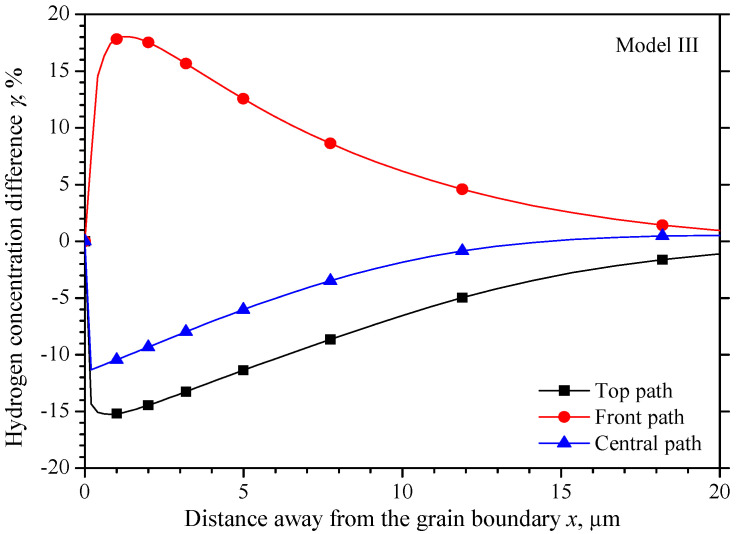
Hydrogen concentration difference equidistant from the grain boundary of model III.

In Figure 13, the maximum hydrogen concentration difference *γ* on the central path locates 0.2 μm equidistant from the grain boundary, with a value of −11.3%, while the maximum differences on the top path and the front path are −15% and 18% within 1 μm away from the grain boundary.

### 5.4. Comparison of the Three Models

Unlike the other two models, the hydrogen distribution in MC-ST bicrystal is symmetric about the *x*-axis, the hydrogen concentration has the same gradient from center to the surface in both *y*-direction and *z*-direction. This is the result of an identic elastic modulus for the MC grain in the *y*-direction and *z*-direction, so as the ST grain. It is obvious that the elastic modulus equals *E*_100_ in both the *y*-direction and *z*-direction in the MC grain. The crystal orientation along *y*-direction and *z*-direction are <121> and <110> respectively in the ST grain. However, the orientation coefficients *k*_110_ and *k*_121_ are both 0.25 according to Equation (16), which leads to an identic elastic module of 193.6 GPa for both *E*_110_ and *E*_121_. So, a similar hydrogen concentration distribution could be found on the top path and the front path in Figure 9.

The MC-MID grain is composed of two misoriented crystals, of which the MC grain has an identic elastic modulus equal to *E*_100_ along the *y*-axis and *z*-axis, while the MID grain has a different elastic modulus of *E*_110_ and *E*_100_ in *y* and *z* directions, respectively. Firstly, focus on the top surface perpendicular to the *y*-axis (top path in Figure 4 and Figure 5), the maximum hydrogen concentration appears in the MID grain with *E*_110_ in the *y*-direction, while the minimum hydrogen concentration locates in the MC grain with *E*_100_ in the *y*-direction. An opposite hydrogen concentration trend could be found with the distance away from the grain boundary, beside the narrow region close to the grain boundary, the hydrogen is higher in the MID grain than the MC grain equidistant from the grain boundary. Moreover, the average hydrogen concentration is 4.50% in the MID grain and −1.24% in the MC grain with 2.73% on the grain boundary. Concerning the surface perpendicular to the *z*-axis (front path in Figure 4 and Figure 5), the hydrogen concentration also has an opposite trend when distance away from the grain boundary. However, the hydrogen concentration in MC grains is much higher than that in MID grains. The average concentration in MC grains is 1.47%, the average concentration in MID grains is −0.52%, and the grain boundaries are −0.91%. By comparing the hydrogen on the top path and front path in the MID grain, the hydrogen concentration is away higher on the top path, which has a larger elastic modulus perpendicular to the surface.

The relationship between the elastic modulus and the hydrogen distribution is not unique in the MC-MID grain, but also the MID-ST grain. The highest hydrogen concentration located on the front surface in the ST grain (on the front path in Figure 11 and Figure 12), and along the *z*-axis perpendicular to the front surface, the elastic modulus of the ST grain is *E*_110_, which is larger than that in the MID grain of *E*_100_. The hydrogen concentration is higher in the ST grain than the MID grain equidistant from the grain boundary, and the average hydrogen concentration is 5.73% and −2.85% respectively with 2.24% on the grain boundary. The elastic modulus is *E*_110_ along the *y*-axis in both grains of the MID-ST grain, but the hydrogen concentration is always lower in the ST grain than in MID grain equidistant from the grain boundary along the top path, with the average hydrogen concentration of −3.71% and 4.17% respectively, and the hydrogen concentration is −2.23% on the grain boundary. Moreover, the hydrogen concentration is away higher on the front path than the top path in the ST grain, which has a larger elastic modulus perpendicular to the surface than its neighbor.

Of all the three misorientation bicrystals along *y*-axis and *z*-axis, no matter the different elastic modulus is caused by the two neighboring grains, or the two directions of one grain, the difference is caused by *E*_100_ and *E*_111_ along the two directions. Now focus on the *x*-direction of the tensile direction, by defining the elastic modulus mismatch ratio as the elastic modulus ratio of the stiff and the softer grains, the elastic modulus ratios are 2.06, 3.20, and 1.55 in the MC-MID, MC-ST, and MID-ST grain respectively. The highest hydrogen concentration differences *γ* in the three models are 21.4%, 29.6%, and 18.0% respectively. A higher elastic modulus ratio will lead to higher hydrogen concentration differences could be found.

Even the hydrogen concentration is quite different equidistant from the grain boundary, they will tend to have a balance value on the grain boundary to keep the continuity of concentration, so there is a large hydrogen concentration gradient very close to the grain boundary, and it is about ±0.2 μm within the grain boundary in this study.

## 6. Conclusions

By establishing a constitutive model of 316 stainless steel crystal anisotropic elastic response, the hydrogen diffusion influenced by crystal orientation near the grain boundary is determined. The results show that hydrogen diffusion near the grain boundary has a great relationship with the grain orientation.

(1) The redistribution of the hydrogen near the grain boundary is driven by the hydrostatic pressure caused by the misorientation of two neighboring grains, the influence is obviously close to the grain boundary, and tends to disappear far from the grain boundary. The hydrogen concentration has a large gradient in a narrow region very close to the grain boundary to balance the concentration difference caused by the misorientation of two neighboring grains.

(2) A higher elastic modulus ratio along the tensile direction will lead to a higher hydrogen concentration difference in the two grains equidistant from the grain boundary.

(3) Beyond the tensile direction, if the elastic modulus is identical along the other two directions in both grains, the hydrogen concentration distribution will be axisymmetric about the tensile direction. If two adjacent crystals have the same elastic modulus in a direction other than the stretching direction, the surface with a larger elastic modulus along the third direction will have a higher hydrogen concentration.

Finally, the proposed constitutive model has a certain guiding significance for the hydrogen diffusion of 316 stainless steel in the grain boundary region; this method will become a general method for studying the hydrogen diffusion between the grains of different kinds of materials.

## Figures and Tables

**Table 1:** Crystal orientations considered for grains.

Grain Type	*x′*	*y′*	*z′*
Most compliant (MC)	[100]	[010]	[001]
Middle (MID)	[1-10]	[110]	[001]
Stiffness (ST)	[111]	[-12-1]	[-101]

**Table 2:** Incompatibility bicrystal models with three types of GBs.

Incompatibility Model	Grain A	Grain B
Model I	MC	MID
Model II	MC	ST
Model III	MID	ST

## Data Availability

All data contained within the article.
